# Development of an Alcohol Refusal Training in Immersive Virtual Reality for Patients With Mild to Borderline Intellectual Disability and Alcohol Use Disorder: Cocreation With Experts in Addiction Care

**DOI:** 10.2196/42523

**Published:** 2023-04-26

**Authors:** Simon Langener, Jan Kolkmeier, Joanne VanDerNagel, Randy Klaassen, Jeannette van Manen, Dirk Heylen

**Affiliations:** 1 Department of Human Media Interaction University of Twente Enschede Netherlands; 2 Centre for Addiction and Intellectual Disability Tactus Addiction Care Enschede Netherlands; 3 Nijmegen Institute for Scientist-Practitioners in Addiction Radboud University Nijmegen Netherlands

**Keywords:** virtual reality, conversational agent, embodied agent, persuasion, peer pressure, addiction, alcohol, intellectual disability

## Abstract

**Background:**

People with mild to borderline intellectual disability (MBID; IQ=50-85) are at risk for developing an alcohol use disorder (AUD). One factor contributing to this risk is sensitivity to peer pressure. Hence, tailored trainings are needed to practice alcohol refusal in impacted patients. Immersive virtual reality (IVR) appears promising to engage patients in dialogs with virtual humans, allowing to practice alcohol refusal realistically. However, requirements for such an IVR have not been studied for MBID/AUD.

**Objective:**

This study aims to develop an IVR alcohol refusal training for patients with MBID and AUD. In this work, we cocreated our peer pressure simulation with experienced experts in addiction care.

**Methods:**

We followed the Persuasive System Design (PSD) model to develop our IVR alcohol refusal training. With 5 experts from a Dutch addiction clinic for patients with MBID, we held 3 focus groups to design the virtual environment, persuasive virtual human(s), and persuasive dialog. Subsequently, we developed our initial IVR prototype and conducted another focus group to evaluate IVR and procedures for clinical usage, resulting in our final peer pressure simulation.

**Results:**

Our experts described visiting a friend at home with multiple friends as the most relevant peer pressure situation in the clinical setting. Based on the identified requirements, we developed a social-housing apartment with multiple virtual friends present. Moreover, we embedded a virtual man with generic appearance to exert peer pressure using a persuasive dialog. Patients can respond to persuasive attempts by selecting (refusal) responses with varying degrees of risk for relapse in alcohol use. Our evaluation showed that experts value a realistic and interactable IVR. However, experts identified lacking persuasive design elements, such as paralanguage, for our virtual human. For clinical usage, a user-centered customization is needed to prevent adverse effects. Further, interventions should be therapist delivered to avoid try-and-error in patients with MBID. Lastly, we identified factors for immersion, as well as facilitators and barriers for IVR accessibility.

**Conclusions:**

Our work establishes an initial PSD for IVR for alcohol refusal trainings in patients with MBID and AUD. With this, scholars can create comparable simulations by performing an analogous cocreation, replicate findings, and identify active PSD elements. For peer pressure, conveying emotional information in a virtual human’s voice (eg, paralanguage) seems vital. However, previous rapport building may be needed to ensure that virtual humans are perceived as cognitively capable entities. Future work should validate our PSD with patients and start developing IVR treatment protocols using interdisciplinary teams.

## Introduction

People with mild to borderline intellectual disability (MBID) are particularly affected by the burden of an alcohol use disorder (AUD). MBID combines the groups mild intellectual disability (IQ=50-69) and borderline intellectual functioning (IQ=70-85). About 15% of the population hold such lower intellectual and adaptive abilities (eg, related to problem solving, abstract thinking, and planning) [1]. Over the past decades, research revealed that people with MBID are at risk for developing an AUD [2,3]. AUD is a spectrum diagnosis ranging from mild to severe, depending on how many of the 11 criteria are met (related to impaired control over quantity and duration of use, social impairment, risky use, tolerance, and withdrawal) [4]. However, despite the high prevalence in addiction care, most treatment programs are not tailored to the diverse needs and limitations of patients with MBID [5], and thus preclude access to effective treatments for this group [2].

Previous research identified multiple factors that are related to relapse, such as limited self-efficacy, coping, and social skills [6]. Self-efficacy can be defined as an individual’s belief in his/her own abilities to achieve a particular goal [7]. In this regard, patients with MBID and AUD are specifically vulnerable to relapse in social contexts, given their susceptibility to peer pressure and lack of adaptive skills to refuse drinking [8-12]. Peer pressure can be divided into 2 types: The first, direct peer pressure, involves (verbal) contact with another person. The second, indirect pressure, arises from observing behavior of a person or group associated with substance use (eg, seeing persons having a party). The psychological working mechanism can be explained using the Theory of Planned Behavior [13]. Accordingly, patients’ attitude to remain abstinent conflicts with the subjective norm to consume alcohol, causing dissonance (ie, psychological stress because of contradictory messages). As a result, conformity toward the subjective norm can emerge to eliminate the internal (ie, attitude) instead of external (ie, violating norm) conflict, as people with MBID and AUD lack self-efficacy (ie, belief in one’s power) to refuse alcohol. Thus, improving self-efficacy seems crucial to avoid conformity behavior in socially susceptible patients.

Hence, training refusal skills to resist peer pressure constitutes a regular practice in drug rehabilitation [14]. For instance, during role-play patients can train and rehearse risk situations to improve their self-efficacy [6]. However, such trainings lack authentic environments to account for indirect peer pressure. Further, trained professionals and other actors are required, which complicates rehearsal and standardization. However, these practical experiences seem essential for treating people with MBID and AUD [15]. Therefore, we are investigating immersive virtual reality (IVR) as a *doing* instead of a talking approach [16] using computer-generated, alcohol-related contexts, in which persuasive embodied conversational agents (ECAs) can be embedded to simulate peer pressure in an authentic and controllable manner [17].

ECAs are digital interfaces with a body (part) that are able to converse with humans using modalities such as speech, gestures, and facial expressions. Several scholars studied social influence via ECAs for (mental) health [18-23]. Further, using virtual humans as a source of persuasive messages has been shown to affect the effectiveness of implemented persuasion strategies [24,25]. These ECAs, however, used mainly informational social influences (the need to be right) [26-31], even though normative influences (the need to be liked) with relational behavior (eg, building rapport [32-38]) appear decisive for peer pressure. Therefore, persuading toward alcohol use will mostly rely on appeals to emotion (ie, logical fallacies) and normative influences (eg, expectations by peers). Further, to enforce conformity, peer leaders should emphasize the violation of group norms [31]. Until now, however, no research explored the persuasiveness of ECAs for IVR peer pressure, nor explored persuasive ECAs for people with MBID [12].

In this study, we aim to establish design guidelines for a behavior change support system to refuse alcohol in patients with MBID and AUD. In doing so, we embedded a persuasive ECA to simulate peer pressure, by following the Persuasive System Design (PSD) model as defined by Oinas-Kukkonen and Harjumaa [39,40]. Through this, we aim to understand active persuasive design elements to avoid blackboxing (ie, lacking understanding of working mechanisms in complex systems) [41]. Considering our ethical responsibility, we initially performed our cocreation with specialized experts for patients with MBID to avoid adverse effects and reduce interaction barriers for our patients. The following research questions were addressed:

- How to design the immersive virtual environment (IVE)?
- How to design the persuasive virtual agent?
  - How to design the virtual agent’s appearance?
  - How to design the persuasive dialog?
- How do the experts experience the IVR with regard to clinical usage?

## Methods

### Cocreation Approach

**Figure 1:**
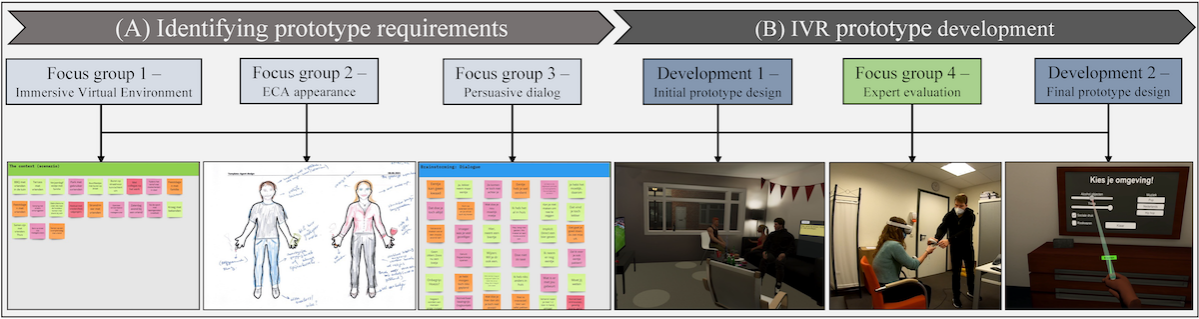
The cocreation approach: (A) Identifying prototype requirements using 3 focus group sessions and (B) the IVR prototype development, along with an expert tryout and focus group to improve our IVR for clinical usage. ECA: embodied conversational agent; IVR: immersive virtual reality.

We followed the PSD model (see below) to specify our system requirements and guide the implementation of related persuasive design features [40]. Building on our theoretical analysis of the persuasive intent, event, and strategy, we conducted 3 focus groups with experts to cocreate the IVR (Figure 1). For this, we focused on the (1) IVE, (2) persuasive ECA, and (3) dialog. Based on the derived requirements, we built our first IVR prototype. We then conducted another focus group with the same experts to evaluate the IVR for clinical suitability and to discuss its potential uses for treatment, considering our ethical responsibility for patients with MBID.

### Participants

Participants were recruited from the staff of a Dutch addiction clinic, specialized in treating individuals with MBID and substance use disorder. Exclusion criteria for our expert evaluation were a history of migraine, epilepsy, motion sickness, severe visual or motor impairment, or being unable to wear the head-mounted display (HMD).

In total, 2 psychologists, 1 psychiatrist, 1 nurse specialist, and 1 psychomotor therapist participated. Experts were all female, had a mean age of 37 (SD 10.3) years, and worked on average since 9.8 (SD 7.2) years in addiction care. The technology experience rating (using a 7-point Likert scale) indicated a high computer (mean 5.20, SD 1.1) know-how compared with a medium videogame (mean 3.20, SD 1.5), as well as low IVR (mean 2.40, SD 1.1) and dialog system (mean 1.80, SD 0.8) knowledge. Most (n=3) had previous experience with IVR for addictive disorders from prior research projects of our group.

### Materials

#### Theoretical PSD

We followed the PSD model by Oinas-Kukkonen and Harjumaa [40,41] for a theoretical analysis of the persuasion context and subsequent integration of persuasive design principles. To understand the persuasion context, we defined the intent of persuasion (ie, persuader, change type), persuasive event (ie, use, user, technology), and persuasive strategy (ie, message, route). Based on the persuasion context, we established our PSD template (Table 1), by integrating suitable persuasive design principles of primary task support (ie, simulation, rehearsal, tunneling, self-monitoring), dialog support (ie, social role), and social *influence* (ie, social facilitation, normative influence).

For primary task support, our IVR provides experience to patients by simulating human-human interactions in a realistic IVE, allowing to link cause and effect during peer pressure. Moreover, it allows to repeatedly rehearse refusal skills by using enactment and multimodal experiences. For dialog support, the ECA simulates a social role for building rapport and exerting social influences. The intended route to persuasion may be described as indirect, targeting emotions, habits, and interpersonal influences. Lastly, a user interface (UI) serves as the primary task support, by tunneling patients through dialogs with our ECA. Here, the UI provides self-monitoring feedback for a better refusal skills acquisition. As described by the PSD model [41], we treat reciprocity as user characteristic that is targeted by the persuader’s appeals.

**Table 1:** Theoretical persuasive system design: toward an IVR^a^ refusal skills training for patients with MBID^b^ and AUD^c^.

PSD^d^ category and design principles			Application level		Implementation
**Primary task support**					
	Simulation	IVR prototype		Simulation of human-human persuasion to enable the observation of cause and effect in realistic settings.	
	Rehearsal	IVR prototype		Rehearsing refusal strategies with an ECA^e^ through enactment and multimodal experiences in IVR.	
	Tunneling	Dialog interface		Dialog UI^f^ guides users through interactions with the ECA in a narrative manner.	
	Self-monitoring	Dialog interface		Dialog UI provides feedback when selecting refusal responses, helping to observe suitable coping behaviors.	
	Tailoring^g^	Customization interface		Customization UI allows customizing the simulation to users’ needs.	
**Dialog support**					
	Social role (likability, similarity)	Virtual agent(s)		Virtual agents simulate an interpersonal relationship with users to exert interpersonal influences realistically.	
**Social *influence*^h^**					
	Social facilitation	Virtual agent(s)		Virtual agents collectively show facilitating behaviors, indicating that others are consuming alcohol along with the user.	
	Normative influence	Virtual agent(s)		Virtual agents elicit normative (peer) pressure to drink alcohol during social gatherings.	

^a^IVR: immersive virtual reality.

^b^MBID: mild to borderline intellectual disability.

^c^AUD: alcohol use disorder.

^d^PSD: Persuasive System Design.

^e^ECA: embodied conversational agent.

^f^UI: user interface.

^g^Implemented after our expert-based evaluation.

^h^Original “social support” may be misleading, hence changed to “social influence.”

#### ECA Prototype and Hardware

For our persuasive ECA, we implemented the customizable Unity Multipurpose Avatar 2 (UMA2) and seated the model within the user’s social space (ie, 2 m). We implemented the Agents United Dialogue Platform (ie, dialog manager, including generation of behavior markup language [BML]) [42], multimodal BML realizer Artificial Social Agents Platform (ASAP) [43,44], and related ASAP-Unity Bridge [45]. This includes Microsoft’s text-to-speech (TTS) engine (ie, Dutch Frank) with body language (ie, lip sync, gestures, gaze), partially scripted using the WOOL platform (WOOL Foundation; ie, gesture, gaze) [42], with gestures and posture animations using Unity’s built-in Mecanim system. For user input, we integrated a dialog UI instead of speech recognition to avoid usability issues, given that the reliability of speech-to-text engines remains troublesome. Hence, we provided buttons with selectable refusal responses based on the identified utterances during our cocreation. Upon selection, a narrator TTS (ie, Flemish Bart) was used to express selected refusal responses to users for a more natural dialog flow.

To demonstrate the IVR prototype, we utilized an Oculus Quest 2 HMD, touch controllers, and a suitable laptop (Core i7-10875H CPU [central processing unit], 16 GB RAM, NVIDIA GeForce RTX 2080 Super) with Oculus Link (via a USB 3.1 cable). We included a virtual hand embodiment and teleport locomotion (with projectile curve) to predefined anchors, with an around 2×2 m room-scale area for physical locomotion. The IVR interactions were built in Unity3D using the XR Interaction Toolkit (preview, version 1.0).

### Measures

For our cocreation, we formulated brainstorming goals based on the 3 major components found in the PSD template: (1) IVE, (2) persuasive ECA(s), and (3) dialog. For (1) IVE, we asked “In what contexts do patients with MBID and AUD face persuasive attempts by another person to drink together?” to identify the 5 most significant contexts. For our (2) persuasive ECA, we asked “How should the persuader in the context of [outcome (1)] look like?” to identify appearance (eg, look, age, gender, body shape, clothing, and properties) and character trait requirements. For this, experts were asked to rapid prototype an ECA using a paper-based template. Lastly, for the (3) dialog, we asked “What are typical arguments of the persuader?,” “What are typical appeals to emotion of the persuader?,” and “What are good, fair, or bad coping reactions by the patient based on the identified appeals” to create ECA interactions. We used the collaboration platform Miro to collect our data.

For our expert evaluation, we created a semistructured focus group with 6 open-ended questions, elucidating the (1) experts’ first impression of the IVR and ECA interaction, that is, (2) user experience, (3) persuasive mechanisms, and (4) persuasive power; (5) procedures for patients with MBID and AUD; as well as (6) experts’ intention to use our IVR for treatment. To support recall, we provided paper-based templates to note down experiences and observations during IVR try out.

### Ethics Approval

Ethics approval was obtained from the University of Twente’s ethics committee (approval number RP 2021-154) and the care institution’s scientific board.

### Procedure

For the cocreation and evaluation, all participants were thoroughly informed, signed the informed consent, and completed a demographic questionnaire. After welcoming participants to the focus group sessions, the audio, screen, and video recordings were started. For cocreation, the researcher explained brainstorming rules and introduced a specific goal for each session. For insights into the research background, a presentation was shown during the first focus group. In subsequent sessions, experts were reminded to adhere to rules and the research background was briefly repeated.

For the expert evaluation, another focus group with the same experts was held 16 weeks later. We invited participants in groups of 2 to evaluate and observe the prototype with the IVR apparatus alternately. First, 1 participant was introduced to the HMD and controllers, while the other one received a paper-based template for taking notes by observing the participant and IVR. Then, a tutorial was conducted to train interactions, by letting the participant use teleport locomotion, customize the virtual hand’s skin tone, and experience the dialog UI without ECA and alcohol cues present. Upon completion, experts were immersed into the enriched IVE via verbal storytelling and asked to engage in a dialog with the ECA by selecting (refusal) responses on the related UI. Finally, experts were asked 5 questions using a Visual Analog Scale (VAS; ie, cravings, ECA persuasiveness, presence, anthropomorphism, and perceived safety), implemented for future patient evaluations. Then, experts alternated, and the procedure was repeated. Upon completion, a focus group was held with all experts using Microsoft Teams to evaluate the IVR prototype toward clinical suitability. The experts received no compensation besides their regular salary.

### Data Analysis

We used the filled-in digital and paper-based templates, along with ranking and agreements during our focus group sessions, to design our IVR. The audio recording from our expert evaluation was transcribed verbatim for subsequent thematic analysis, with the aim of identifying requirements for our IVR development. We applied the reflexive protocol by Braun and Clarke [46,47], by using the researcher’s theoretical sensitivity and reflexivity, to analyze experiences of experts when interacting with our IVR. For this, the researcher SL followed the 6-step protocol: For (1) data familiarization, SL listened to the recording and (re)read the transcript. Subsequently, (2) relevant segments in our data were identified, coded, and collated. Afterward, (3) initial (sub)themes were generated using our coded data and (4) reviewed by revisiting the themes with respect to data set and goal. Lastly, (5) final themes were defined and our (6) report was produced. For this, we used the qualitative data analysis program ATLAS.ti (version 9.1.6; ATLAS.ti Scientific Software), and themes were discussed among participating researchers (ie, SL, JV, and RK) during refinement (steps 4 and 5) to eliminate discrepancies within the analysis.

## Results

### Overview

In the following section, we describe our (1) findings from the cocreation with experts in addiction care, and (2) prototype development with subsequent expert evaluation to tailor IVR and procedures toward clinical applications.

### Findings From Our Cocreation With Experts in Addiction Care

#### Description of Findings

The following paragraphs describe the major findings from our focus groups with experts per IVR component: (1) IVE, (2) persuasive ECA, and (3) dialog.

#### Identifying Persuasion Context and IVE Design

In the first focus group, experts agreed on risk settings in which patients with MBID and AUD are persuaded to consume alcohol, with the most significant identified being *visiting a friend at home with multiple friends.* Design requirements include a small social-housing apartment with kitchen and living room. The IVE should be designed dark, noisy, and messy. The living room should comprise a TV (streaming a soccer match); couch; loud Dutch music; and a table with snacks, bottles (with falling over noises), and cigarettes or shag. Multiple friends should sit on the couch under the influence of alcohol, holding a beer or cigarette, talking loudly (eg, laughing, shouting), and asking users to drink or provide alcohol by default.

#### Designing a Persuasive ECA for Eliciting Peer Pressure

In the second focus group, experts defined requirements for our persuasive ECA design. Here, experts described a middle-aged (30-40 years), white male with average morphological characteristics (ie, body shape, height, body hair, relaxed posture) and slight paunch. Appearance characteristics include short, unkempt blonde or brown hair and an (week old) unshaved beard. The clothing includes a (too) large black T-shirt (including a brand name), black or gray jogging pants, and sneakers. Other properties named were a small arm tattoo, holding a cigarette, and beer can/bottle from (low-priced) domestic breweries. Besides detailed descriptions, experts agreed that ECA(s) should be realistic but not detailed regarding personal attributes when no customization (ie, based on user input) was possible. Yet, ECA(s) should have a nonchalant, slightly bent posture and amicable personality when sitting on the couch next to the patient.

#### Constructing a Persuasive Dialog With Refusal Responses

In the third focus group, experts reported on persuasive utterances and related refusal responses. Before persuading patients toward drinking, ice-breaking and rapport building should be integrated, such as greetings (eg, “Hey, nice that you are here”), well-being questions (eg, “How are you?”), or providing comfort (eg, “Get out of your coat and make yourself at home”). As assumed, ECA utterances should comprise primarily appeals to emotion (eg, “You used to be much more sociable”) and habits (eg, “That is what you always do”) in contrast to logic or reason. Furthermore, based on identified persuasive utterances, experts reported low (eg, “No man, I stopped”), fair (eg, “Not now”), and high-risk (eg, “Yes, one will be okay”) responses.

### Prototype Development and Evaluation—Toward an IVR Refusal Skills Training

#### Description of Findings

Following the 3 focus groups, we developed a prototype based on the identified requirements. In the following sections, we describe our initial design, prototype evaluation with the experts, and refined simulation based on the evaluation outcomes.

#### IVR Prototype Development Based On the Cocreation With Experts

**Figure 2:**
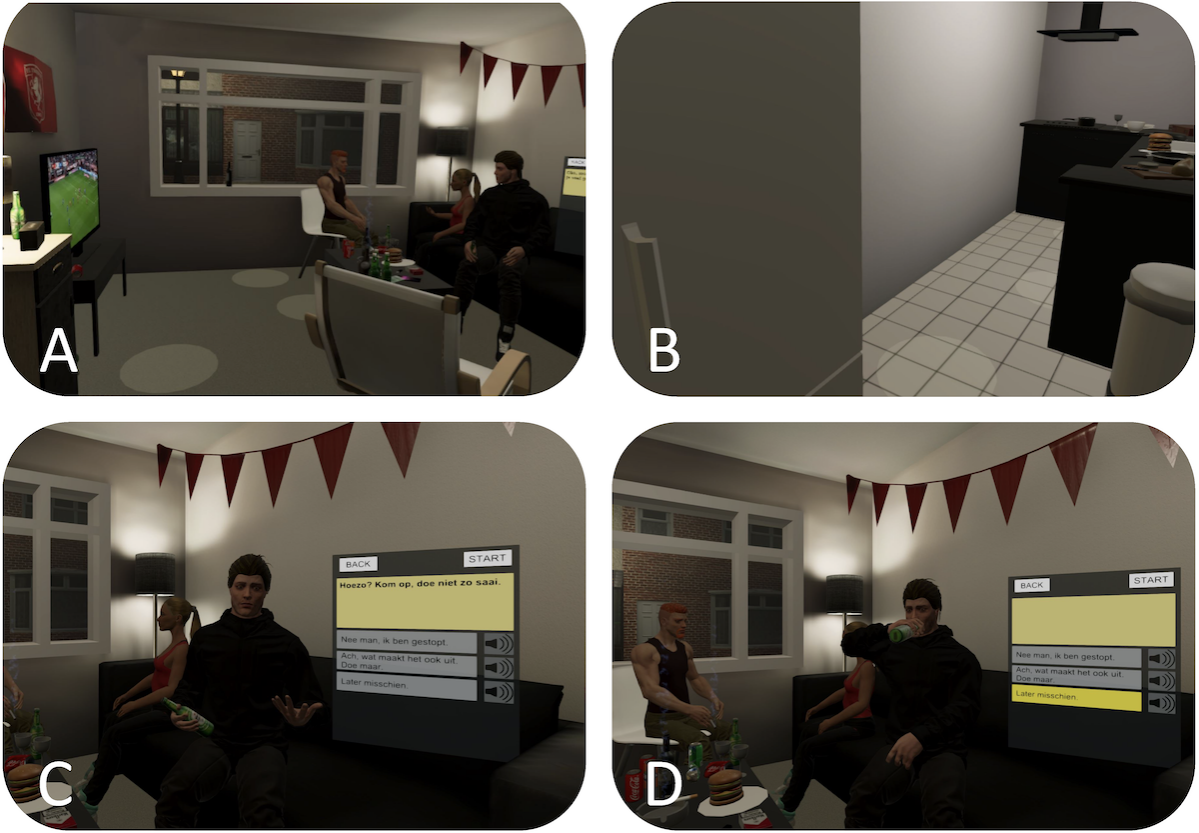
The initial prototype: (A) living room with agents sitting on a couch/chair, alcohol-related cues, TV streaming soccer, and wireless speaker shuffling Dutch party music; (B) small kitchen with interactable fridge to grab beer or liquor; (C) ECA persuading toward drinking; and (D) dialog UI to selected refusal responses of different riskiness. ECA: embodied conversational agent; UI: user interface.

Based on our cocreation, we developed a (1) social-housing apartment in IVR (Figure 2A and 2B) with a kitchen and a living room. Further, we integrated a couch with friends under the influence of alcohol, TV streaming a soccer match, and loud Dutch music via a virtual wireless speaker. To achieve a realistic and plausible IVE, alcohol cues (eg, fridge, beer, wine, liquor) and related objects (eg, cigarettes, shag with paraphernalia, darts, football emblem, flags, food) were added. Users were able to open the fridge/cabinet and manipulate objects (eg, darts, bottles). For our (2) persuasive ECA, we created a male in his mid-30s with unkempt hair, week-old beard, and beer bottle in hand (Figure 2C). Moreover, we dressed the ECA with a black hoodie, jogging pants, and sneaker for a universal look. For the (3) dialog, we implemented 5 consecutive levels (Multimedia Appendix 1): building rapport and ice-breaking, appeal to habit, first persuasive attempt, second persuasive attempt, and closing consequence. Finally, we implemented a dialog UI that provides traffic light feedback when selecting refusal answers to learn adequate behaviors (Figure 2D).

#### Expert-Based Evaluation

##### Themes Overview

After developing our prototype, we conducted an expert-based evaluation with the same participants; 5 themes emerged to tailor IVR and related procedures toward clinical usage: (1) Design Factors for IVR Immersion, (2) Design Features for Peer Pressure ECA(s), (3) User-Centered Customization, (4) Behavior Change Support, and (5) Design Features for IVR Accessibility.

##### Design Factors for IVR Immersion—Multimodality, Interactivity, and Narratives

Regarding immersion factors, we found the subthemes *multimodality*, *interactivity,* and *narratives.* For *multimodality*, we identified visual (ie, real-life congruence, animations) and auditory (ie, music, background noise) qualities as immersive (“Yes, and it is indeed true that with the music, it helps to be highly immersed.” [E3]), while olfactory cues, despite adding realism, should be treated with caution to avoid adverse effects in users. Nonetheless, our IVR’s *interactivity* creates plausible scenarios (“But also the number of actions you could do just made it feel real, and also the mess on the table and stuff. It felt relative to other worlds I’ve seen less clean and artificial, so to speak.” [E5]), which could be enhanced by adding *narratives*, as experts suggested to start with a tutorial to learn basic controls, followed by an invitation to a party, and transition to a dressing room to customize the simulation (“...do that storytelling in the tutorial setting, that it is also included there. Because then you will be more immersed.” [E3]). Moreover, to improve real-life congruence, increasing messiness (eg, dirty spots, clothes), adding extra alcohol cues (eg, wine, booze, a crate of beer, glass with liquid inside), sounds (eg, football match), and diverse background agents (eg, older age, obesity) talking with audible voice were suggested.

##### Design Features for Peer Pressure ECA(s)—Insulting Dialog, Alcohol-Use Animations, and Voice Vividness

For our persuasive ECA design, we found the subthemes *insulting dialog*, *alcohol-use animations*, and *voice vividness*. For *insulting dialog*, the ECA’s intrusive, insistent, and alcohol offering behavior was described (“..., he was going on and on the whole time [...] and also saying some mean things. It’s realistic, I guess, I’m afraid.” [E5]). Yet, group dynamics (“...and nudging each other a little bit like: ‘He is not using at all.’...Two against one.” [E5]) and rapport building were named to improve the simulation. For *alcohol-use animations*, experts found drinking/offering behavior to contribute to the perceived persuasiveness, though animations were described as somewhat unnatural (“...I had to get used to the woodiness a bit.” [E5]). Lastly, for *voice vividness*, experts identified lack of paralinguistic features when using TTS engines, such as intonation and stress (“That is the only thing I thought of: ‘well, that makes it unrealistic now’. And I think that specifically intonation is very important for our group.” [E1]). Hence, experts suggested to replace the TTS by voice recordings to boost the persuasive power.

##### User-Centered Customization—Personal Goals, Tailorable Simulations, and an Incremental Difficulty

For user-centered customization, we found the subthemes *personal goals*, *tailorable simulation*, and *incremental difficulty*. Here, the patient’s *personal goals* should be linked to specific IVR interventions (“There are in fact many different goals, which as far as I’m concerned, you can link to this.” [E4]). For assessment, experts suggested an initial exploration (ie, craving, bodily signals) and creation of a “crisis alert plan” based on risk situations. For treatment, experts elucidated the usefulness of IVR for body-centered learning (eg, bodily signals, coping through mindfulness), self-reflection (eg, cause and effect), or usage as a confrontation tool (eg, for dangerous self-overestimation). For this, *tailorable simulations* are needed, to customize the IVR to the patient’s needs (ie, messiness, drinks, agents, music). Further, an *incremental difficulty* is required, by using a step-by-step approach, to avoid adverse effects in patients (“Because you want to give people cravings and they have to feel completely immersed into that situation, but it doesn’t have to become so real that people really get into trouble.” [E5]). Ideally, trainings should be designed for an error-free learning in people with MBID (“...it is also important to say that clients with disabilities should initially practice error-free with maximum amount of help.” [E5]).

##### Behavior Change Support—System- and Therapist-Delivered Interventions

Two strategies for behavior change support were identified: *system-delivered* and *therapist-delivered support*. For *system-delivered support*, formative (eg, dialog-related feedback) and summative feedback (eg, closing praise) should be provided. However, regarding formative feedback, experts were inconclusive about how to display it, either as constant active, after utterance selection, or deactivated, suggesting different difficulty levels when used (“...then indeed you get a little bit of, this is a right answer, and this is a wrong answer.” [E3]). Nonetheless, all experts agreed that IVR therapy should be *therapist delivered* to learn and reflect upon bodily signals and coping (“In fact, you want them to practice the situation and then you can engage in dialogs about ‘What happened?’ and ‘So what did you think about that?’” [E3]). Instead of talking about the past, patients can talk about the present, making abstract concepts (eg, cravings) graspable (“You always talk about something that someone has experienced in the past, with which you try to prepare them for something again in the future. So then, your talk therapy is actually always a little bit out of this world.” [E5]). Lastly, experts elucidated that timing (ie, mid till end of treatment, self-overestimation) and frequency (ie, multiple sessions) depended on personal goals, but were discordant about talk therapies while patients (and therapist) are immersed in IVR.

##### Design Features for IVR Accessibility—Facilitators and Barriers

Lastly, for an accessible IVR design, experts identified 3 *facilitators* and *barriers*. For *facilitators*, experts described realistic interactions, such as walking and grabbing objects (“The walking, you can see well, and I also liked the tutorial.” [E1]). Here, limiting teleport locomotion to anchors was not perceived as restriction and to learn the controls, the tutorial was described as vital element. Further, the virtual hands served as visual and spatial feedback, by observing animations (“I did like that you, so to speak, really see what you’re doing when grabbing.” [E3]). Yet, controller scaffolding was described helpful by just 2 experts (“Found it useful that it was there. Just: ‘oh yes, that button was for grabbing.’ A kind of confirmation.” [E2]), as it was not noticed by the others. Lastly, the use of a VAS was rated positive, for instance, as a “thermometer” to measure tension. Still, experts encountered *barriers* when using text-based procedures (“...if that text written out, if that doesn’t distract, in some way, from what you’re doing.” [E5]). Hence, VAS should avoid displaying text to the user (eg, ask items verbally) and ECA utterance should be removed from the UI. Further, to tailor the system to the user’s needs, the terminology used should be simple instead of abstract (eg, *walk* instead of *teleport*). Lastly, experts described minor interaction barriers (eg, opening the fridge while grabbing objects) and suggested using a buffer zone to avoid sudden proximity when teleporting (“That’s unnatural then. You stand so close; you wouldn’t stand like that in real life.” [E2]).

#### Final Prototype Design Based on the Prototype Evaluation

**Figure 3:**
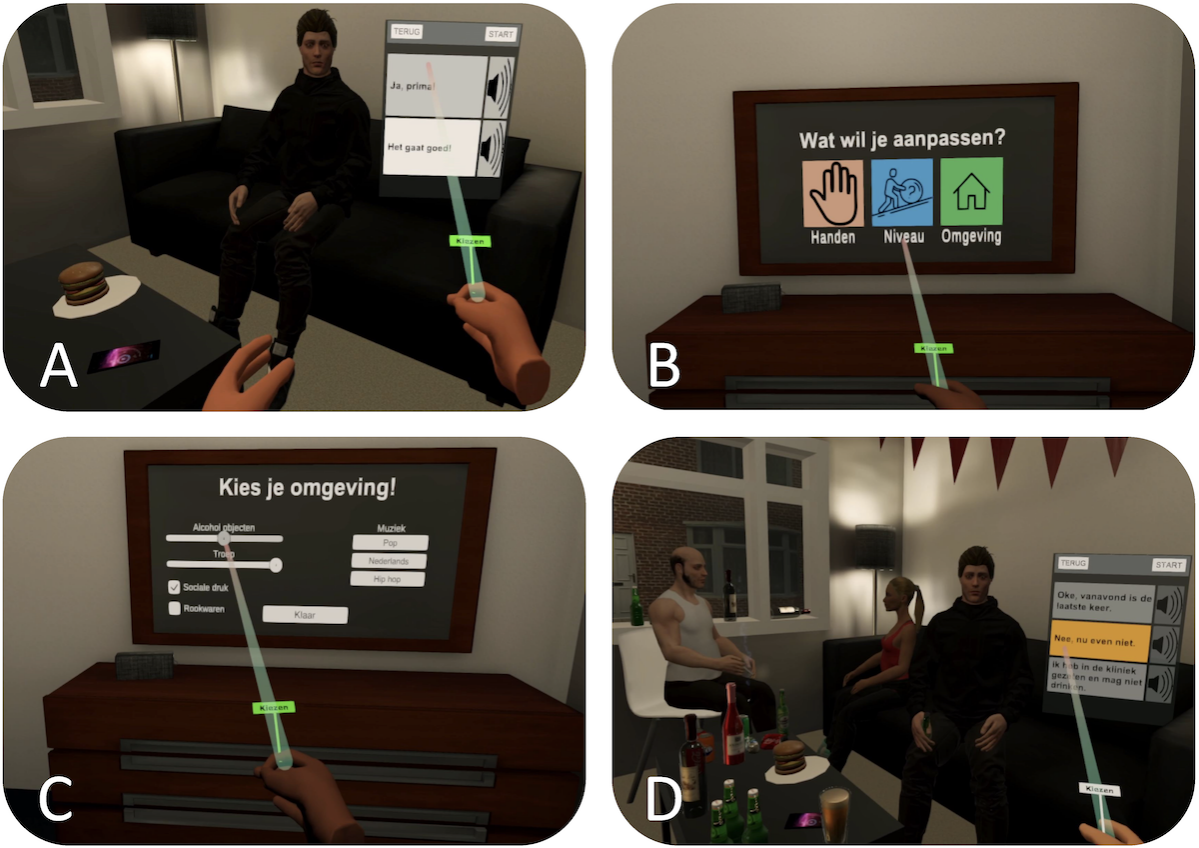
The final IVR prototype with narrative immersion: (A) friend inviting to a party this evening; (B) dressing room to customize the IVR simulation (ie, virtual hands skin tone, difficulty, IVE); (C) IVE customization (ie, alcohol cues, messiness, social pressure, smoking cues, music); and (D) persuasive ECA interaction using refusal responses of different riskiness levels. ECA: embodied conversational agent; IVE: immersive virtual environment; IVR: immersive virtual reality.

After the expert evaluation, we improved our prototype design and immersion procedures for future evaluations in patients with MBID. To amplify the immersion and stimulate rapport building with the ECA, we implemented a narrative immersion using storytelling instead of introducing scenes verbally. The patients start in the tutorial and receive an invitation to a party this evening (Figure 3). Users then go upstairs to a dressing room to customize the IVR, including virtual hands (ie, skin tone), difficulty (ie, system-delivered feedback), and IVE (ie, cues, messiness, social pressure, music). Patients are then immersed into the enriched IVE, with other guests, alcohol-related cues, and related objects. For a better usability, we reduced text to a minimum, improved the terminology with simpler language, and added a buffer zone when using teleport to anchors. Further, the system-delivered feedback (ie, traffic light) can be deactivated to allow for unrestricted therapist-delivered interventions.

## Discussion

### Principal Findings

This work reports on the cocreation of an IVR alcohol refusal training for patients with MBID and AUD. Following our theoretical PSD, we conducted 3 focus groups with experts from a specialized addiction clinic for patients with MBID to understand the persuasion context and establish design guidelines. Based on the identified requirements, a first peer pressure simulation was developed. Then, we evaluated the IVR with the same experts to tailor prototype and procedures toward clinical usage. For our IVR design, experts described the risk situation of *visiting a friend at home with multiple friends*. Based on the data, we developed a social-housing apartment with alcohol-related objects and intoxicated virtual humans present. To simulate peer pressure, we embedded a virtual man with generic appearance who persuades patients with MBID to drink alcohol. Patients can practice alcohol refusal skills with the ECA by selecting responses from a dialog UI. Following the playtesting, experts described the implemented multimodality, interactivity, and narratives as factors for deep immersion. For peer pressure, the ECA’s insulting dialog and nonverbal animations (eg, offering alcohol) were described as contributing design features. To advance the ECA’s persuasiveness, however, improving *voice vividness* (eg, paralinguistics) and adding group dynamics were suggested. Still, the IVR should remain customizable (ie, IVR, training difficulty) based on the patient’s personal goals. For behavior change support, experts preferred therapist over system-delivered interventions to avoid a perilous try-and-error in patients with MBID. Lastly, facilitators (ie, embodied interactions, tutorial, VAS) and barriers (ie, text-based procedures, complex terminology) for IVR accessibility were described.

For our IVE design, experts described multimodality, interactivity, and narratives as important factors to immerse patients with MBID. Here, we used our prior experience in developing IVR interactions for this group, by designing a small but realistic IVE with embodied interactions, anchor-based teleportation, and controller scaffolding [48]. Previous work reported such error-free and positive user experience as a major factor leading to user satisfaction [41,49]. Similar to our prototype, other scholars built alcohol-related IVEs for exposure purposes [50]; however, to elicit realistic behavioral responses, plausible interactions are required [51]. Hence, compared with others, we advanced IVR interactions on the object/conversation level and integrated a narrative to engage users realistically with our tutorial and subsequent refusal skills training. Interestingly, narratives might act as persuasive feature [52], presumably as part of *simulation* [41]. Previous work showed that narratives can influence behavioral intentions for health behaviors [52]. Hence, influencing and experiencing the own narrative appear interesting for IVR therapy.

For our ECA, we established a first PSD to avoid blackboxing [53], allowing to identify active PSD elements and replicate findings in diverse user and use areas. For our cocreation, we looked into the ECA and persuasive dialog to connect specific design elements to our PSD. Here, we systematically designed for the *social role* of a virtual friend to transfer emotional appeals that induce realistic reactions (eg, guilt) in patients. Previous studies on IVR for AUD embedded mostly “nonintelligent” agents as social cues [54-56]. Yet, as learning paradigms appear promising for IVR addiction therapy [54], interactable and persuasive ECAs are needed to simulate peer pressure, a factor well-known to cause relapse [2]. Using our IVR enables novel treatments using *direct peer pressure*, but also adds realism to *indirect peer pressure* during virtual reality therapy.

Nonetheless, experts described our ECA as low in (persuasive) power due to a lack in *voice vividness*. Similar findings were observed when persuading with anger [31], though this was dependent on power relations. This is fascinating, given that power dimensions appear similar to group dynamics in peer pressure (ie, group leaders exhibit greater power [31]). Thus, presumably ECAs must be seen as a cognitive entity with social power to convey appeals to emotion/habit (*normative influence*) in IVR [57]. Our findings suggest that this may be achieved through the body’s (non)verbal communication channels [58], such as *voice vividness* (ie, paralinguistics) and animations (eg, drinking). Further, to establish social power, narratives may be useful to build rapport with ECAs, while multiagent interactions (eg, 2 against 1) could establish social hierarchies.

Lastly, for clinical usage, experts preferred therapist- as opposed to system-delivered interventions to explore symptoms, stimulate self-reflection, and train coping skills [59,60]. Similar findings were reported by Skeva et al [59], who described the gain of self-efficacy during relapse prevention trainings as the key factor for control in reality. For this, we assume that *rehearsal* may be refined by letting individuals express utterance themselves instead via a narrator voice, while keeping the feature for accessibility. Lastly, we found that Skeva et al [59] emphasized cognitive-behavioral practitioners to engage with IVR, while particularly vulnerable groups could benefit from body-centered paradigms. In our evaluation, we found that disciplines would use this tool differently [59], for instance, with focus on bodily signals during psychomotor therapy. Thus, future research should create IVR therapy protocols using interdisciplinary teams to establish a taxonomy with learning goals [54,61]. Further, system-delivered approaches should not be neglected, for example, as homework [59], as IVR therapy is often seen as adjunct treatment.

### Limitations

The findings of this study have to be seen in light of some limitations: First, we cocreated our IVR prototype by involving specialized professionals from an addiction clinic, therefore, perception in patients with MBID and AUD might differ, given the preconditioned behavioral patterns in AUD. Hence, patient evaluations are required to validate the ECA’s persuasiveness, including replication studies using our template. Our PSD enables scholars to develop similar experiences through an analogous user-centered design using our ECA architecture. Further, scholars should learn from our unimplemented requirements, such as (intra)group dynamics (eg, multiagent scenarios) and natural voices with paralinguistics, to induce peer pressure. Second, we have not yet explored ECA animations in a systematic manner. Instead, we used animations for a natural interaction. Hence, nonverbal communication forms a promising factor for *normative influence* that should be addressed in future work, although it was not missed by our experts. For this, scholars should consider facial expressions [31], gaze, and gestures, presumably combined with natural speech to create plausible interactions [51]. Lastly, we implemented a traffic light feedback as part of the dialog UI to make *self-monitoring* discussable. However, future research should systematically explore error-free training approaches that make use of formative and summative feedback.

### Future Work

Future work should clarify factors that enable ECAs to elicit peer pressure in IVR. Our findings suggest that paralinguistic and nonverbal information form vital factors for persuasion toward alcohol use in patients with MBID and AUD. Hence, subtle emotional expressions, along the verbal appeals, seem to take a crucial role for persuasion using virtual humans, which connotes to further explore interpersonal (eg, rapport, group dynamics, interpersonal stance) and empathetic (eg, facial expressions, social touch) behaviors along the aforementioned information using natural speech. For rapport building and social hierarchies, integrating narratives seems auspicious, for instance by playing short games with ECAs before starting refusal trainings. Thus, future research with patients should be performed to further investigate specific PSD elements and validate the IVR for future work, in particular to develop therapy protocols with interdisciplinary teams. Lastly, peer pressure via ECAs should be studied more comprehensively to understand the power in influencing humans toward negative behavior, including potential usage for therapy, ethics to adhere to, and possible negative effects on therapy adherence (within outpatient settings).

### Conclusions

Our findings establish a PSD for IVR peer pressure simulations in patients with MBID and AUD. Using an analogous design approach, researchers can replicate findings and explore effective PSD features for peer pressure ECAs. Throughout our cocreation with experts, we learned that appeals to emotion/habit should be conveyed via natural human speech channels (ie, verbal and nonverbal). For this, subtle emotional information must be provided via paralinguistics in the ECA’s speech (eg, intonation), gestures (eg, handing over beer), and interpersonal dynamics (eg, group formation, rapport effects). For behavior change support, experts favored therapist-delivered interventions to avoid a perilous trial and error. Future work should further investigate factors contributing to persuasive power in virtual humans, such as emotional information in speech and nonverbal expressions to induce realistic peer pressure. Further, research with interdisciplinary teams should be conducted to establish IVR therapy protocols with specific pedagogical learning goals for our patients.

## Appendix

Multimedia Appendix 1 Persuasive dialog and coping strategies using tunneled behavioral feedback in a traffic light manner.

## Abbreviations

ASAP: Artificial Social Agents Platform
AUD: alcohol use disorder
BML: behavior markup language
ECA: embodied conversational agent
HMD: head-mounted display
IVE: immersive virtual environment
IVR: immersive virtual reality
MBID: mild to borderline intellectual disability
PSD: Persuasive System Design
TTS: text-to-speech
UI: user interface
UMA2: Unity Multipurpose Avatar 2
VAS: Visual Analog Scale

## Data Availability

The data supporting the findings of this work are available from the corresponding author (SL) upon reasonable request.
