# Supplementary figures and images for: Development of an Alcohol Refusal Training in Immersive Virtual Reality for Patients With Mild to Borderline Intellectual Disability and Alcohol Use Disorder: Cocreation With Experts in Addiction Care
Source: JMIR Form Res. 2023 Apr 26;7:e42523. doi: 10.2196/42523 (PMC10173034; doi:10.2196/42523)

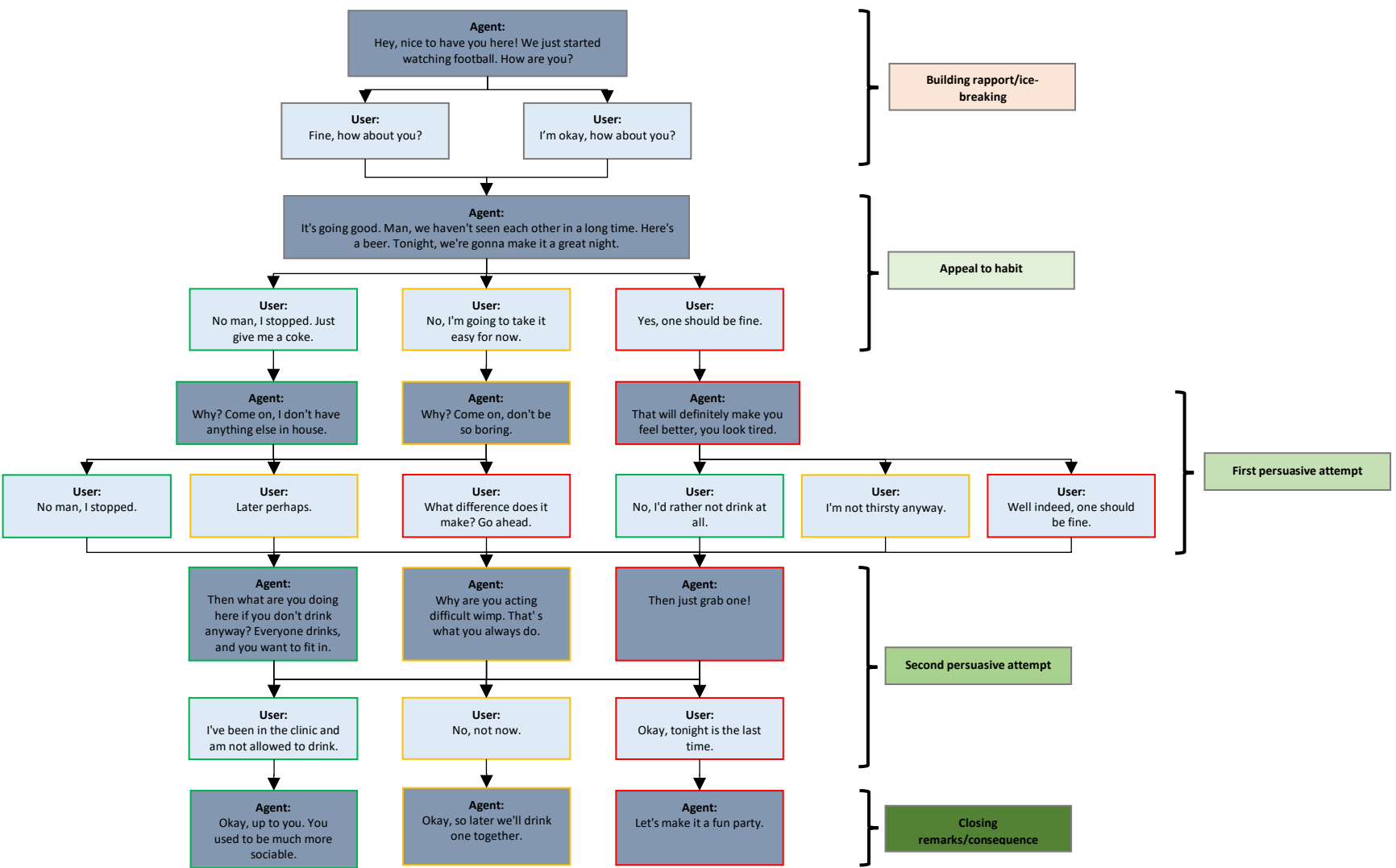

Supplement: Multimedia Appendix 1 [file formative_v7i1e42523_app1.pdf]
